# Supplementary material for: The Systems Biology Research Tool: evolvable open-source software
Source: BMC Syst Biol. 2008 Jun 29;2:55. doi: 10.1186/1752-0509-2-55 (PMC2446383; doi:10.1186/1752-0509-2-55)
Supplement: Additional file 1 — SBRT Archive. An archive of the current version of the Systems Biology Research Tool. [file 1752-0509-2-55-S1.zip › sbrt-1.4.0/doc/developers_guide/api/sbrt/shell/text/AppInputFileLineFormat.html]

AppInputFileLineFormat


|  |  |  |
| --- | --- | --- |
| **Overview** \| **Package** \| **Class** \| **Use** \| **Tree** \| **Deprecated** \| **Index** \| **Help** | |  |
| **PREV CLASS**   **NEXT CLASS** | **FRAMES**    **NO FRAMES**     **All Classes** |  |
| SUMMARY: NESTED \| FIELD \| CONSTR \| METHOD | DETAIL: FIELD \| CONSTR \| METHOD |  |


---


## sbrt.shell.text Interface AppInputFileLineFormat

**All Superinterfaces:**: Format, MapFormat<java.lang.String,java.lang.String>

**All Known Implementing Classes:**: AppInputFileLineFormatVersion1

---

``` public interface AppInputFileLineFormat extends MapFormat<java.lang.String,java.lang.String> ```

This interface is used to represent the format of
lines in process input files.

**Author:**
:   This interface was written and documented by
    Jeremiah Wright while in the Wagner lab.

**See Also:**: `ProcessInputFile`

---

**Method Summary**

**Methods inherited from interface sbrt.shell.text.MapFormat**

`formatKey, formatKeys, formatPair, formatPair, formatValue, formatValues, formatValues, parseAllKeys, parseAllPairs, parseAllValues, parseAllValues, parseKey, parsePair, parseValue`

---


|  |  |  |
| --- | --- | --- |
| **Overview** \| **Package** \| **Class** \| **Use** \| **Tree** \| **Deprecated** \| **Index** \| **Help** | |  |
| **PREV CLASS**   **NEXT CLASS** | **FRAMES**    **NO FRAMES**     **All Classes** |  |
| SUMMARY: NESTED \| FIELD \| CONSTR \| METHOD | DETAIL: FIELD \| CONSTR \| METHOD |  |


---
